# *Leuconostoc mesenteroides* periprosthetic knee infection, an unusual fastidious Gram-positive bacteria: a case report

**DOI:** 10.1186/s12879-017-2315-y

**Published:** 2017-03-23

**Authors:** Rafael Franco-Cendejas, Claudia A. Colín-Castro, Melissa Hernández-Durán, Luis E. López-Jácome, Silvestre Ortega-Peña, Guillermo Cerón-González, Samuel Vanegas-Rodríguez, Jaime A. Mondragón-Eguiluz, Eduardo Acosta-Rodríguez

**Affiliations:** 1 Infectious Diseases Department, Instituto Nacional de Rehabilitación Luis Guillermo Ibarra Ibarra, Av. México-Xochimilco #289 Col. Arenal de Guadalupe. Del. Tlalpan, Mexico City, CP 14389 Mexico; 2 Hip and Knee Reconstruction Department, Instituto Nacional de Rehabilitación Luis Guillermo Ibarra Ibarra, Av. México-Xochimilco #289 Col. Arenal de Guadalupe. Del. Tlalpan, Mexico City, CP 14389 Mexico

**Keywords:** *Leuconostoc mesenteroides*, Periprosthetic joint infection, Biofilm, Case report

## Abstract

**Background:**

Periprosthetic joint infections are mainly caused by Gram-positive cocci. *Leuconostoc mesenteroides* is a rare microorganism mainly causing bloodstream infections. At times, it might be confused with another type of cocci and give rise to misdiagnosed infections. Molecular diagnosis and biofilm production comprise important techniques to guide antibiotic treatment.

**Case presentation:**

A 68-year-old Hispanic female with a previous history of bilateral knee arthroplasty presented with acute right-knee inflammation and gait impairment. Blood tests showed inflammatory response and knee x-ray revealed no prosthesis loosening. Irrigation and debridement was performed. Gram-positive cocci were obtained from cultures, and then biochemical and molecular identification revealed *L. mesenteroides*. Susceptibility and biofilm production were performed. The patient was treated with IntraVenous (IV) Ceftriaxone for ten days and was then switched to Amoxicillin-Clavulanate for 3 months with clinical and laboratory success.

**Conclusions:**

Microbiology diagnosis of fastidious microorganisms is mandatory to treat periprosthetic joint infections adequately. *L. mesenteroides* may infect non-immunocompromised persons; however, treatment guidelines are lacking.

## Background

*Leuconostoc* species are Gram-positive microorganisms with coccoid morphology and catalase-negative reaction [1]. *Leuconostoc* spp. have been involved in a variety of infections [2–7], particularly in patients being treated with Vancomycin and in immunocompromised patients; in addition, they have also been documented in outbreaks [8]. Species within this genus are not easily identified in clinical microbiology laboratories, with clinical isolates sometimes being reported incorrectly as *Enterococcus* species or *Streptococcus* species by routine biochemical testing. The distinction among these different bacteria may exert an important impact in selecting the appropriate antibiotic regimen for the patient’s treatment, because all clinical isolates of *Leuconostoc* spp. possess high-level resistance to Vancomycin, with a Minimal Inhibitory Concentration (MIC) >256 μg/mL [9].

Prosthetic Joint Infections (PJI) comprise a significant part of acute and chronic infections in many hospitals and are related with clinical and social problems, such as prolonged hospitalization and frequent, additional surgical procedures, which are associated with an increased risk of hospital complications and prolonged antimicrobial treatment [10, 11]. These infections are principally linked with the surgical procedure or with an infection either near to the prosthesis or elsewhere. Germs that commonly infect these devices are those belonging to the genus *Staphylococcus*. Moreover, a wide range of bacteria responsible for PJI is able to produce biofilm on prosthetic implants. Biofilm is a complex microbial environment protected by a self-produced polymeric matrix and it adheres to various surfaces [12]. Its formation notably hinders sampling and culturing, because traditional sampling techniques are not able to detach biofilm-embedded bacteria from prosthetic surfaces, thus leading to negative results [13]. There are clinical guidelines for treating this type of infections; these recommend debridement and retention strategy in patients with fewer than 3 weeks of symptoms onset [14]. Patients who do not comply with this criterion should undergo a single-stage or 2-stage exchange strategy depending on their clinical condition and should receive antibiotic therapy according to antimicrobial susceptibility.

To date, a periprosthetic joint infection caused by *L. mesenteroides*, to our knowledge, has yet to be described.

## Case presentation

**Fig. 1:**
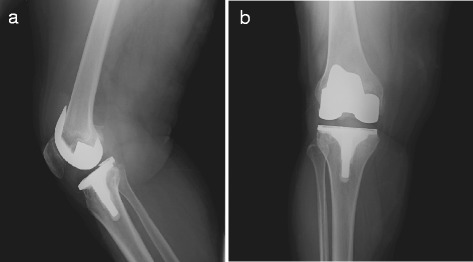
Preoperative x-ray images of the **a**) lateral and **b**) AnteroPosterior (AP) view of the right knee showing prosthesis without loosening

**Fig. 2:**
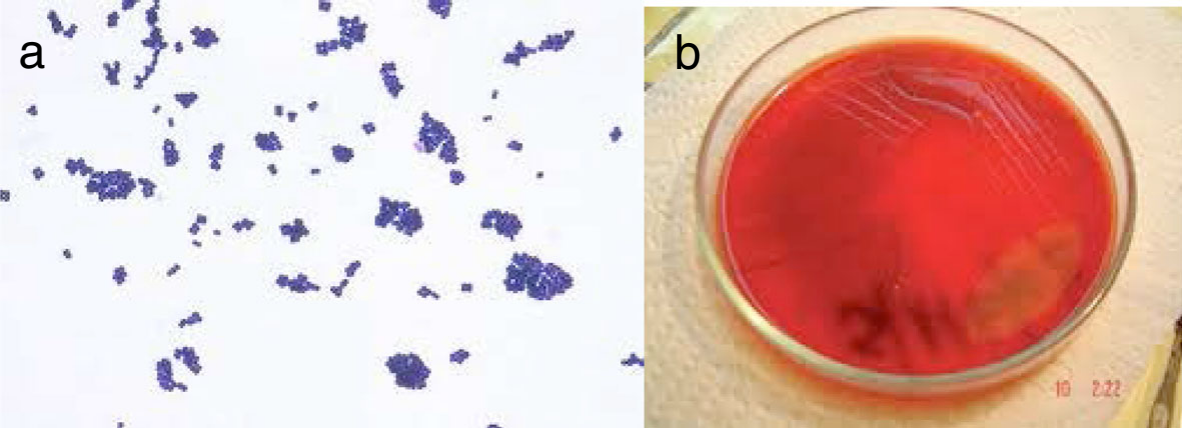
**a**) Gram stain showing Gram positive cocci. **b**) Typical white and small bacteria colonies on 5% sheep blood agar

A 68-year-old Hispanic female, with a previous diagnosis of arterial hypertension on Enalapril treatment, obesity with BMI of 35 and bilateral knee osteoarthritis. The patient received left primary total knee arthroplasty in December 2008, and right primary total knee arthroplasty in March 2010. She had an upper respiratory tract infection 3 weeks prior to hospital admission without receiving any antibiotic therapy. She debuted in March 2013 with right knee erythema, edema, local hyperthermia, and increasing pain on engaging in any type of movement. On admission, the patient had a fever of 38.2 °C, blood pressure of 110/80 mmHg, and a pulse rate of 96 beats/min, she denied taking antibiotics or having food poisoning previously. Laboratory examinations revealed a hemoglobin level of 14.5 g/dL, a platelet count of 281,000/ lL, and a white-cell count of 25,400/lL, and C-Reactive Protein (CRP) of 65 mg/dL (0–6 mg/dL). A plain knee radiograph showed gas absence or prosthesis loosening (Fig. 1). Arthrocentesis puncture was performed and Gram-positive cocci were seen; however the cell count could not be performed because the sample coagulated. Surgical debridement was performed, finding the presence of pus and necrotic tissue at vastus medialis; as well as curettage to obtain bloody weaving and properly consistency at the periphery of the prosthesis, which did not present loosening. Once observed revitalized tissue, three litters of saline solution with gentamicine were irrigated. The liner was changed. Three samples of periprosthetic tissue were obtained and transported immediately to the laboratory. All samples showed Gram-positive cocci in the initial Gram stain. The biopsy specimens were inoculated in conventional culture media and, after 24 h, there was the growth of an organism. The colonies were white, raised, and small on 5% sheep blood agar (Fig. 2). Identification was carried out using the VITEK®2 (bioMérieux, Marcy L’Etoile, France) system, which yielded the result of a *L. mesenteroides*. Biochemical tests were performed as recommended in the literature [15]; briefly, we conducted confirmation of resistance to Vancomycin on Mueller–Hinton agar; gas production was observed from glucose, which did not grow in bile esculin media and which presented arabinose and mannitol assimilation. The broth microdilution susceptibility assay was carried out in Mueller–Hinton agar with lysed horse blood employing the American Type Culture Collection (ATCC) strain 29,213 as positive control; the latter exhibited susceptibility to Penicillin and Ampicillin with MIC 0.062 μg/mL and 0.5 μg/mL, respectively. 16S recombinant RNA (rRNA) gene sequencing was performed on bacterial cultures. DNA was extracted using the QIAmp DNA Minikit method (Qiagen, Ltd., West Sussex, UK) according to the manufacturer’s instructions. Amplification of the bacterial *16S rRNA* gene was performed by real-time PCR in a Light-Cycler instrument (Roche Diagnostics, Mannheim, Germany). The DNA sequence was determined with a sequence analyzer (ABI PRISM 3130xL; Applied Biosystems) employing the same primers as those with the BigDye kit (Applied Biosystems). The sequences obtained were compared with bacterial sequences in the GenBank database utilizing the BLAST program. This analysis demonstrated the microorganism as *L. mesenteroides.* (GenBank: KU517836).1 We evaluated biofilm production by carrying out the Christensen method, revealing a mild level of biofilm production [16].

The patient received treatment with Ceftriaxone 2 g OD for 10 days IntraVenously (IV) due to the lack of a supply of Ampicillin at the time, and was then switched to oral Amoxicillin-Clavulanate 875/125 mg tid for 3 months, when the patient had not knee symptoms and CRP reached 5 mg/dL, without having any side adverse events. Physical therapy was administered for 2 months, with the patient regaining leg strength. Clinical and laboratory evolution was good at 6-month time lapses for 3 years without the presentation of inflammatory knee symptoms and with CRP levels of 3 mg/dL.

## Discussion

We present here a case of acute hematogenous infection of knee prosthesis associated with *L. mesenteroides* 3 years after implantation. The patient’s main symptoms were knee pain and fever. The origin of this microorganism might be the patient’s previous upper respiratory tract infection, which caused hyperpermeability, and the subsequent bacterial entrance into the bloodstream. The microorganism grew in all samples obtained, indicating a high bacterial content in the tissues and on the device. *L. mesenteroides* is a Gram-positive cocci that is a non-producer of catalase reaction, which is sometimes misinterpreted with members of the *Streptococcus* genus. It is widely distributed in nature. At the industrial level, it is used in the production of wines, cheeses, dairy products, and sugar. Infections have been commonly described in patients with immunosuppression and in patients in the Intensive Care Unit (ICU), causing bloodstream infections.

Conventional identification in the laboratory is complex; thus, the use of biochemical and molecular biology techniques is worthwhile to assure a proper diagnosis. Importantly, this genus is intrinsically resistant to Vancomycin associated with chromosomal resistance; therefore, beta-lactams are the preferred antimicrobial to treat this type of infections, e.g., Ampicillin. The patient received Ceftriaxone IV as the first part of the treatment during the 10-day hospital stay, and was then switched to Amoxicillin-Clavulanate for 3 months, the patient exhibiting good tolerance, compliance and complete functional articular recovery.

Although this *Leuconostoc* strain demonstrated biofilm production, it was classified as a mild biofilm producer. Leathers et al. compared different strains of *L. mesenteroides* and found that the biofilm formation capacity varied widely between the strains, and that this ability is related with the amount of its components [17]. There are, to our knowledge, no studies evaluating the activity of Amoxicillin-Clavulanate properties against biofilm production in *L. mesenteroides*; however, in other genera, such as *Staphylococcus* spp., it does not possess activity [18]. The patient did not receive any other drug against biofilm production and, for the 3-year follow-up period, there has not been an infection relapse.

According to the Practice Guidelines of the Infectious Disease Society of America (IDSA) the recommended treatment is 2–6 weeks of pathogen-specific IV and switching to highly bioavailable oral antimicrobial therapy for 3 to 6 months, as well as monitoring for clinical and laboratory response [14]. Amoxicillin-Clavulanate has been considered an alternative antibiotic option in these guidelines, and it should be prescribed three times daily due to its bone pharmacokinetics properties [19]. Surgical debridement must be performed as soon as possible in order to remove all damaged tissue, as was carried out in this patient, because this patient presented symptoms of fewer than 3 weeks and sought attention soon after the device was retained, as suggested.

This case is interesting and useful first because every PJI is important for identifying and for ruling out when it presents acutely in a previously asymptomatic patient, in that surgical debridement is a crucial measure for successfully retaining the device in order to cleanse the periprosthetic tissue and to take samples of different tissues to identify the causing microorganism. The patient did not receive any type of medical care while she experienced the upper respiratory tract infection; thus, it is important to seek medical evaluation if a patient complains of gastrointestinal, urinary, or respiratory infectious symptoms and if the patient has a joint device. The oral antibiotic therapy duration lasted until she was symptoms-free and the CRP reached normal values, as it is suggested for microorganisms different to Staphylococci [14]. Second, as in many other diseases, multidisciplinary collaboration is an essential part of the care of these types of infections. Therefore, it is recommended that PJI be treated at referral hospitals that have all of the services needed, even in low-income countries.

Third, adequate microbial identification is required to provide proper treatment during hospitalization and afterward. Semi-automatized laboratory equipment is helpful in the vast majority of strains, as was observed in this case. However, it is strongly suggested to confirm the diagnosis by another method in order to assure identification and to perform the suitable susceptibility tests, especially cases of fastidious microorganisms, which as in the present case. The knowledge of some microbial features, such as biofilm-production intensity, may afford more information concerning possible treatment success or the addition of another drug against its formation.

This is, to our knowledge, the first report of a Prosthetic Joint Infection (PJI) in an immunocompetent patient that was caused by *L. mesenteroides* with successful treatment.

## Conclusion

We present a case of a *Leuconostoc mesenteroides* periprosthetic knee infection in an immunocompetent woman. An accurate diagnosis is mandatory to avoid antibiotic mistreatment. Debridement surgery and antimicrobial therapy are the pillars of high success in acute PJI. It is highly recommended that patients with symptoms suggesting infection elsewhere seek care in order to decrease the possibilities of infectious prosthetic complications. To date, there are, to our knowledge, no guidelines for treatment that have been described in this type of bacterial infections.

## Abbreviations

AP: AnteroPosterior
BMI: body mass index
CRP: C-reactive protein
ICU: Intensive Care Unit
OD: once day
PJI: Prosthetic Joint Infections
tid: three times daily
